# Preoperative ultrasound radiomics analysis for expression of multiple molecular biomarkers in mass type of breast ductal carcinoma in situ

**DOI:** 10.1186/s12880-021-00610-7

**Published:** 2021-05-17

**Authors:** Linyong Wu, Yujia Zhao, Peng Lin, Hui Qin, Yichen Liu, Da Wan, Xin Li, Yun He, Hong Yang

**Affiliations:** 1 Department of Medical Ultrasound, The First Affiliated Hospital of Guangxi Medical University, Nanning, Guangxi Zhuang Autonomous Region 530021 People’s Republic of China; 2 GE Healthcare, Shanghai, People’s Republic of China

**Keywords:** DCIS, Molecular biomarkers, Radiomics, Ultrasound

## Abstract

**Background:**

The molecular biomarkers of breast ductal carcinoma in situ (DCIS) have important guiding significance for individualized precision treatment. This study was intended to explore the significance of radiomics based on ultrasound images to predict the expression of molecular biomarkers of mass type of DCIS.

**Methods:**

116 patients with mass type of DCIS were included in this retrospective study. The radiomics features were extracted based on ultrasound images. According to the ratio of 7:3, the data sets of molecular biomarkers were split into training set and test set. The radiomics models were developed to predict the expression of estrogen receptor (ER), progesterone receptor (PR), human epidermal growth factor receptor 2 (HER2), Ki67, p16, and p53 by using combination of multiple feature selection and classifiers. The predictive performance of the models were evaluated using the area under the curve (AUC) of the receiver operating curve.

**Results:**

The investigators extracted 5234 radiomics features from ultrasound images. 12, 23, 41, 51, 31 and 23 features were important for constructing the models. The radiomics scores were significantly (P < 0.05) in each molecular marker expression of mass type of DCIS. The radiomics models showed predictive performance with AUC greater than 0.7 in the training set and test set: ER (0.94 and 0.84), PR (0.90 and 0.78), HER2 (0.94 and 0.74), Ki67 (0.95 and 0.86), p16 (0.96 and 0.78), and p53 (0.95 and 0.74), respectively.

**Conclusion:**

Ultrasonic-based radiomics analysis provided a noninvasive preoperative method for predicting the expression of molecular markers of mass type of DCIS with good accuracy.

**Supplementary Information:**

The online version contains supplementary material available at 10.1186/s12880-021-00610-7.

## Background

Breast ductal carcinoma in situ (DCIS) is a kind of malignant tumor originated in the ductal epithelial tissue, limited to the basement membrane [1]. DCIS is the second most common breast tumor, and accounts for approximately 20–30% [2]. Some DCIS had the potential to further develop into breast invasive cancer [3]. The clinical treatments of patients with DCIS include surgical resection, radiotherapy, chemotherapy and endocrine therapy, in which surgical resection includes simple focal resection and mastectomy, with different therapeutic effects [4]. Although the prognosis of DCIS is good, more than 14% of DCIS patients may develop invasive cancer without treatment within 10 years [5]. In the past 10 years, the incidence of DCIS has gradually increased, highlighting the understanding the importance of DCIS pathology [6]. However, the pathologic mechanism of the transition from DCIS to invasive carcinoma is still unclear, which produces clinical challenges of overdiagnosis and overtreatment in patients with DCIS [7]. Therefore, the investigators thought more studies were need to understand the potential of the pathological process of DCIS, in order to adapt to the current individualized, refined treatment.

Immunohistochemistry (IHC) can reflect the expression of molecular biomarkers in tumor tissue, which can further clarify the biological behaviors of tumors. The expression of different molecular biomarkers can lead to different biological behaviors and treatments. Some studies have shown that some molecular biomarkers were important indicators for predicting biological behavior and judging follow-up treatment in patients with DCIS, such as estrogen receptor (ER), progesterone receptor (PR), human epidermal growth factor receptor 2 (HER2), Ki67, p16, and p53. ER and PR are the earliest molecular biomarkers of breast cancer. They are predictors of breast cancer prognosis and endocrine adjuvant therapy [8]. HER2 is a proto-oncogene, which is mainly involved in tumor signal transduction and cell proliferation. Its positive expression can lead to a high distant metastasis rate and poor prognosis of breast cancer. Ki67 is an antigenic nuclear protein that can be used as a proliferation marker. Its high expression is considered to be a biomarker of tumor invasion [9]. Ki67 has a good application prospect in predicting endocrine therapy response of breast cancer [10]. Defined as a tumor suppressor gene, p16 is considered to be an important cell cycle regulator [11]. P16 is closely related to abnormal methylation initiation. P53 is a common tumor suppressor gene. Impaired function of p53, such as p53 mutation, can lead to uncontrolled proliferation of damaged cells [12]. Therefore, accurate identification of the expression of molecular biomarkers can help stratify tumor risk and facilitate the development of personalized and accurate treatment plans.

Currently, the preoperative evaluation of the molecular biomarkers of DCIS mainly depends on IHC detection after biopsy. However, because the progression of tumors are dynamic process, there are differences in spatio-temporal evolution. In addition, the evaluation results of a few tissue biopsies do not necessarily represent the expression of the molecular biomarkers of the whole tumor [13]. Invasive procedures and potential risks limit its multiple applications in monitoring tumor progression and biological behavior. However, the preoperative monitoring of molecular biomarkers can dynamically identify the progression of tumors and the changes in biological behavior, which has great significance for the accurate formulation of treatment plans and the evaluation of curative effects. To avoid overdiagnosis and overtreatment of patients with DCIS, it is necessary to provide dynamic and accurate evaluation of biological behavior information for the clinic.

With the breakthrough of imaging technology, mammography is an important examination method for DCIS, which is sensitive to the detection of calcification [14]. Ultrasound (US) has become the main examination technology to detecting breast lesions [15], which is real-time, dynamic and non-invasive. There are two types of DCIS: mass and non-mass. Some studies suggested that the detection rate of US in 93 patients with mass type DCIS reached 77.4% [16]. The main characteristics of DCIS in ultrasound were: uneven low or slightly low echo, irregular shape, unclear borders, parallel skin, weakened posterior echo, calcification, and some blood flow signals [17, 18]. However, mammography or US-assisted screening could increase overdiagnosis because both tests primarily detect low-grade invasive cancers [19]. At the same time, radiologists are very time-consuming to accumulate experience and have strong personal subjectivity, which is another problem that needs to be solved. There is an urgent need for more advanced imaging evaluation methods to guide the diagnosis and treatment of DCIS.

Breast lesions are diagnosed and screened by various imaging methods, such as mammography, US, and magnetic resonance imaging (MRI). All three examination have some limitations [20]. Radiomics is a hot subject of artificial intelligence that is applied in the medical imaging field, which is the cornerstone of precision science in the future. Radiomics is defined as the extraction of high-throughput features from single or multiple medical image patterns to select features that are closely associated with tumors,and the ultimate goal is to construct prediction models based on features to provide accurate tumor phenotypic analysis information and accurate treatment decision-making [21]. Radiomics highlights the image features that are not visible to the naked eye, thus significantly enhancing the predictive power of medical imaging [22]. Radiomics has been developed in a wide range of fields, such as disease diagnosis and biological behavior judgment. For example, the US-radiomics model developed by Luo WQ et al. had better performance in distinguishing breast lesions than breast imaging reporting and data system (BI-RADS) [23]. Lin F et al. found that the radiomics score was more effective than the clinical radiological model in benign and malignant breast lesions (< 1 cm) [24]. These series of studies showed that radiomics had better performance than traditional imaging features in the diagnosis of breast diseases to some extent.

Thus, this retrospective study intended to further clarify the relationship between US-radiomics and molecular markers of DCIS. Radiomics models had been developed to noninvasively evaluate the expression of molecular markers to help achieve accurate risk stratification and treatment for patients with DCIS.

## Materials and methods

### Study cohort

Clinical data of 400 patients with DCIS who were pathologically confirmed by surgery were retrospectively analyzed by the investigators. The data were based on the pathology reports of the first affiliated hospital of Guangxi medical university from January 2015 to July 2020. Further inclusion and exclusion criteria for this cohort study were as follows. Inclusion criteria: (1) primary breast DCIS, (2) IHC results of molecular biomarkers; and (3) preoperative US data within one month. Exclusion criteria: (1) non-mass DCIS, including manifestations of ductal dilation, diffuse calcification, and diffuse distribution of lesions; (2) unclear image of target lesions; (3) secondary DCIS or postoperative recurrence of DCIS; (4) preoperative treatment history of radiotherapy, chemotherapy and traditional Chinese medicine; and (5) lack of clinical data.

**Fig. 1:**
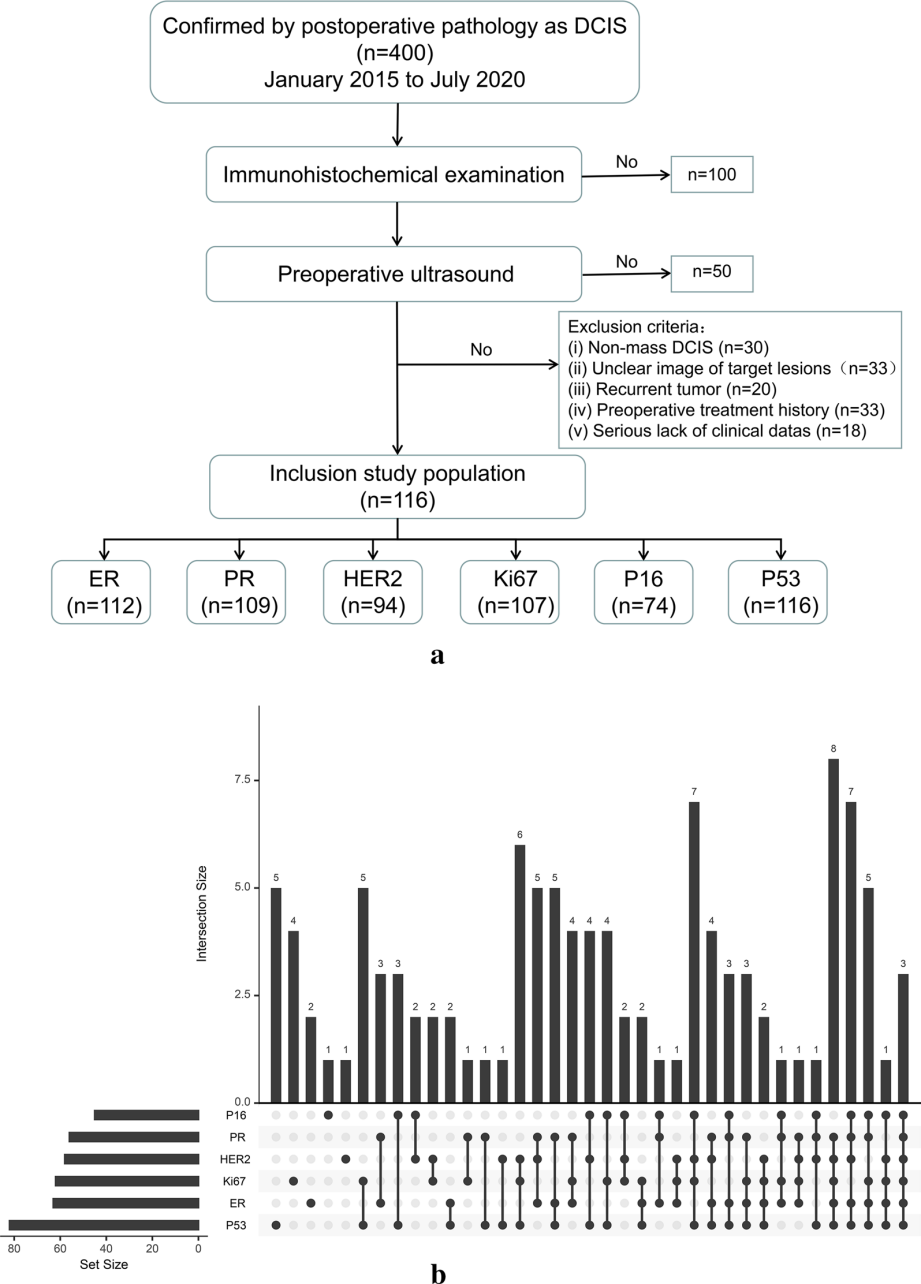
Study cohort. **a** Workflow of study cohort inclusion. **b** Up-set plot of the expression of molecular markers shared between different samples

This study finally enrolled a total of 116 patients with DCIS. The IHC (ER, PR, HER2, Ki67, p16, and p53) conformed to the diagnositic criteria of the department pathology in this hospital, and were classified as positive or negative. The IHC results of Ki67 were positive or high expression (Ki67 >  = 14%) and negative or low expression (Ki67 < 14%) [25]. The number of patients enrolled for each molecular biomarker were listed as follows: 112 cases (ER), 109 cases (PR), 94 cases (HER2), 107 cases (Ki67), 74 cases (p16), and 116 cases (p53) (Fig. 1).

### Image collection and tumor segmentation

Each US radiologist involved in image collection had over 5 years of experience in the field of breast. Before collecting US data, all radiologists were strictly trained. GE Logiq E9 (GE Healthcare, United States), Aloka EZU-MT28-S1 (Aloka, Japan) and MYLAB CLASS C (MYLAB, Italy) medical ultrasound diagnositic instruments were utilized for image collection. The breast probe was selected, and the frequency was set to 7–14 MHz. The patients took the supine position, put their hands on the head, and fully exposed the breast area and armpits on both sides. The lesions were scanned from multiple angles, and the largest clear image of the lesions were selected. The following ultrasonic characteristics of the lesions were recorded: BI-RADS classification, location, size, shape, boundary, internal echo, calcification, posterior echo changes, ductal dilatation, blood flow signal distribution and axillary lymph nodes.

These images were imported into the ITKSNAP software (version 3.8.0). To avoid subjective compliance, two radiologists with five years of working experience manually delineated the region of interest (ROI) of the lesions. The radiologists disregarded the diagnosis and pathological results of the patients [26]. After the discussion, when there was a big difference between the two radiologists, the third radiologist with 10 years of experience re-examined and confirmed the final boundary. This process provided reliable DCIS area contours and ensured the accuracy of feature extraction.

### Image pre-processing and feature extraction

**Fig. 2:**
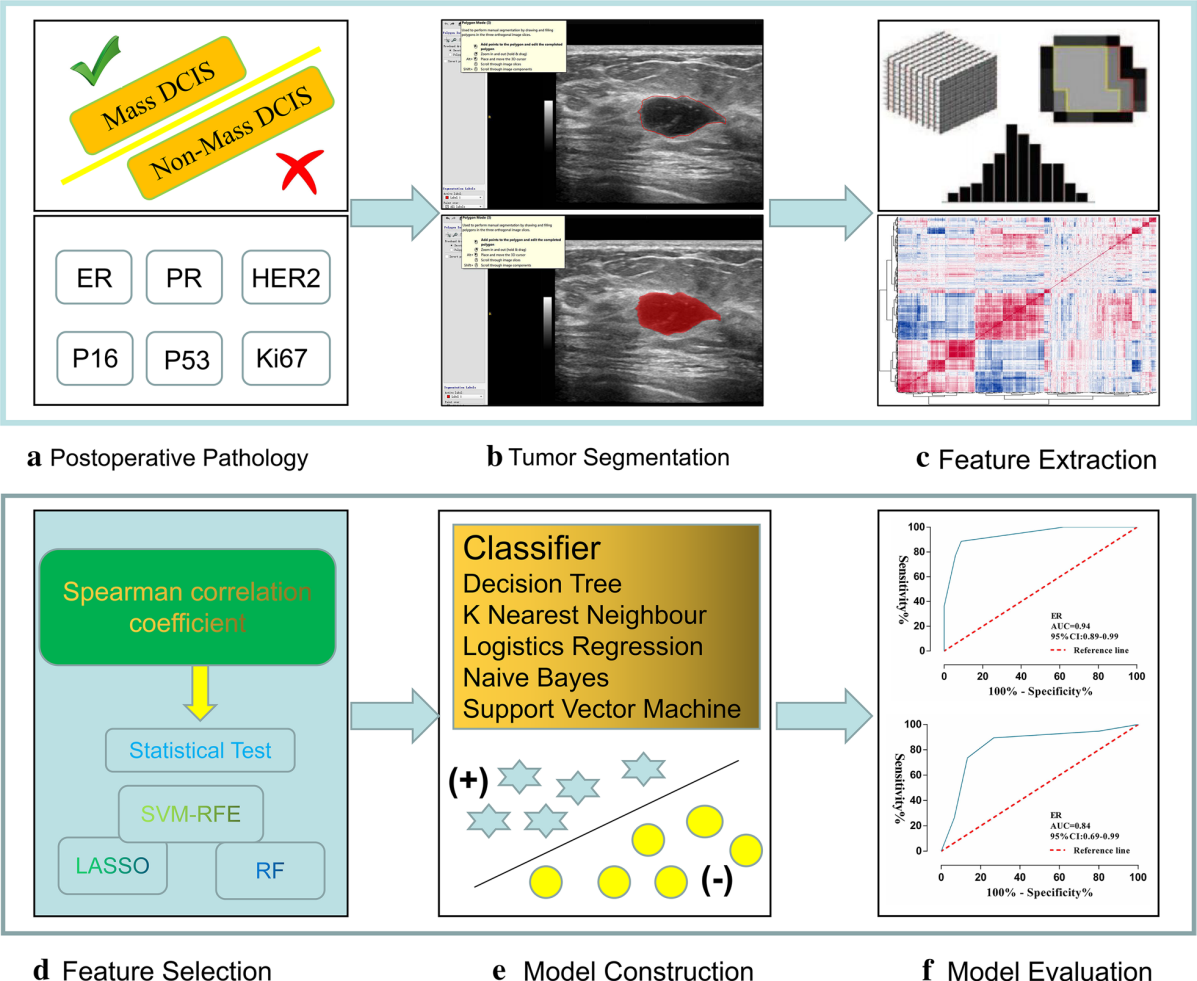
Workflow of radiomics analysis

**Fig. 3:**
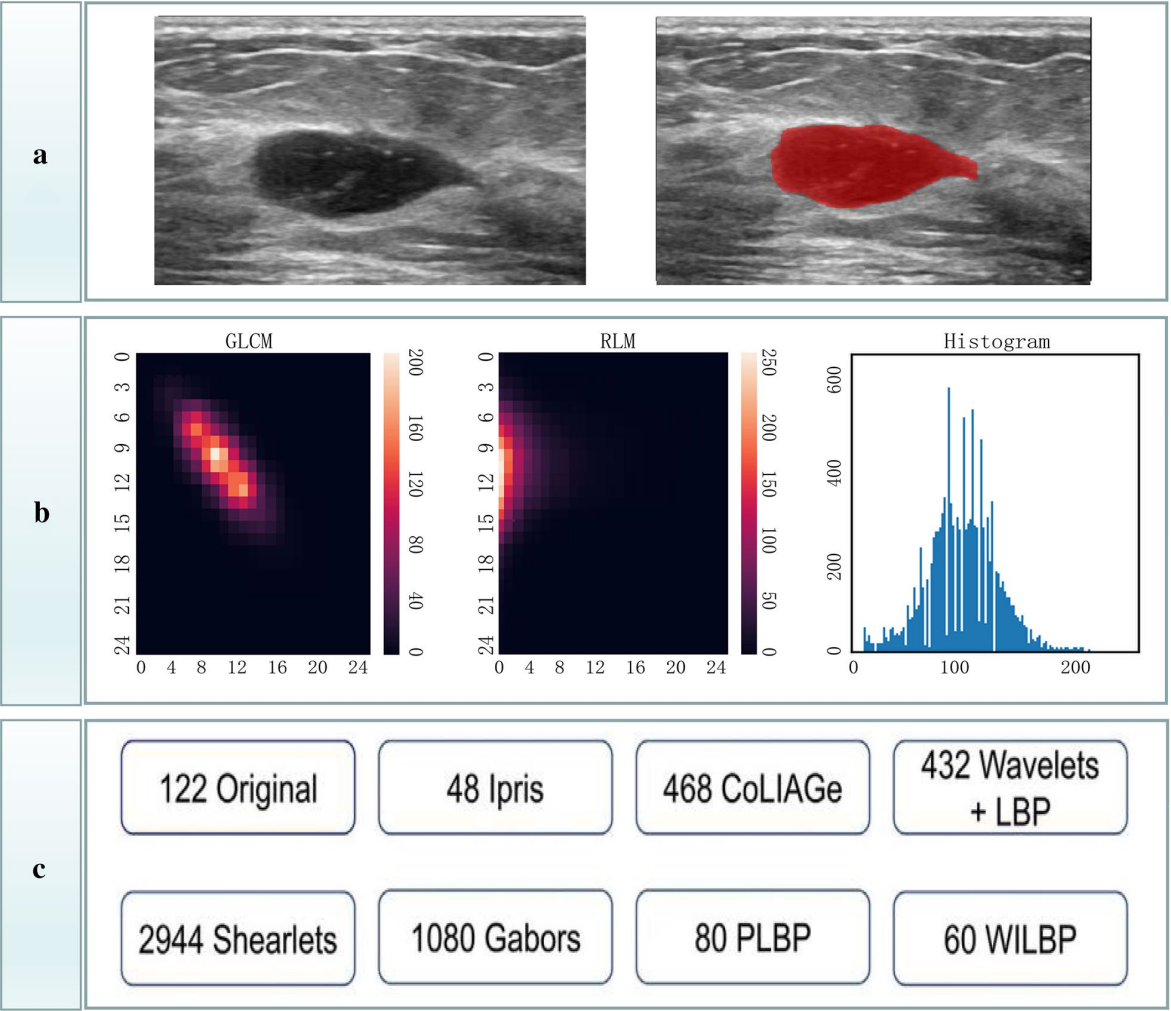
The process of quantifying features. **a** Delineation of the ROIs. **b** Gray level co-occurrence matrix (GLCM), run length matrices (RLM), and histogram feature extraction. **c** The classification of 5234 features

The Intelligence Foundry software (version 1.3, GE Healthcare, Shanghai, China) was applied for radiomics analysis. Figure 2 summarized the main flow of radiomics analysis. The software relied on algorithms provided by the Pyradiomics package that comply with the image biomarker standardization initiative (IBSI, version 2016) [27]. Features were automatically calculated and extracted by the Pyradiomics extractor. The maximum number of features extraction of the software was 5234, including: 122 original, 48 intraperinodular textural transition (ipris), 468 co-occurrence of local anisotropic gradient orientations (CoLIAGe), 432 wavelets + local binary pattern (LBP), 2,944 shearlets, 1,080Gabors, 80 phased congruency-based local binary pattern (PLBP) and 60 wavelet-based improved local binary pattern (WILBP) features (Additional file 1). Before feature extraction, the images were pre-processed: the gray value of the image was discretized with a bin size of 256, and the original features were extracted. The features of wavelets + LBP, Shearlets, Gabors, PLBP and WILBP were extracted by wavelet transform, shearlet transform and garber operator transform on the gray value matrix of the original images, respectively [28] (Fig. 3).

### Data grouping and data cleaning

To balance the initial distribution of data, each sub-data set was randomly split into training set and test set in a ratio of 7:3. Based on the difference in the image extraction feature quantization caused by different medical ultrasound diagnositic instruments and parameters, the combat method was employed to solve this problem. The combat method could be used to coordinate and correct the differences between different machines and different center images. Some studies had applied this method to the MRI images [29]. In addition, the median value of the feature quantization value was applied to fill the missing sample. The min–max normalization method was employed to normalize the feature data to improve the comparability between features. It converted the original data to the range of [0, 1] by linearization, which realized the proportional scaling of the original data.

### Feature importance

The purpose of the study is difficult to explain with thousands of radiomics features of high-dimensional data. Feature importance analysis helps to explain the importance of features for subsequent model constructing. Multiple combination techniques were applied to explain the importance of features: First, Spearman correlation coefficient test was used to eliminate high correlation features with threshold values (0.75, 0.85, 0.95). This test was a statistical index to measure the correlation between two variables. Three dimensionality reduction methods (least absolute shrinkage and selection operator (LASSO) [30], random forests (RF) [31], and support vector machine-recursive feature elimination (SVM-RFE) [32]) separated or jointed statistical tests for selecting the important features. In the statistical test, if the data accorded with the normal distribution, the t-test was adopted; otherwise, the Mann–Whitney U test was adopted.

### Predictive radiomics models

Machine learning algorithms were developed based on Python environment. Five machine-learning-based classifiers (decision tree (DT), k-nearest neighbors (KNN), logistics regression (LR), naive Bayes (NB), and support vector machine (SVM)) were employed to predict the expression levels of the molecular biomarkers of DCIS [33, 34], and the score of each model was calculated. In addition, the fivefold cross-validation method was explored to improve the accuracy of the models. The test set was used to evaluate the reliability of the models.

To accurately evaluate the predictive ability of radiomics models, the receiver operating curve (ROC), the area under the curve (AUC), accuracy (ACC), precision (PREC), sensitivity (Sn) and specificity (Sp) were adopted for the evaluation. The closer the AUC was to 1, the higher the diagnostic efficiency was. In this study, only the best classification results of the classifier were shown.

## Results

### Patient characteristics and molecular biomarkers of interest

The mean age of all patients was 48.8 ± 11.1 years, and the age range was 29–84 years. The characteristics parameters were shown in Table 1. The ultrasonographic features of the patients were similar to those reported in the literatures. The expression of ER, PR, HER2, Ki67, p16, and p53 were as follows: 49 patients with ER-negatives and 63 patients with ER-positives; 53 patients with PR-negatives and 59 patients with PR-positives; 36 patients with HER2-negatives and 58 patients with HER2-positives; 45 patients with Ki67-negatives and 62 patients with Ki67-positives; 29 patients with p16-negatives and 45 patients with p16-positives; 34 patients with p53-negatives and 82 patients with p53-positives.

**Table 1:** Patient characteristics and molecular biomarkers of interest

Parameters	N = 116	Parameters	N = 116
Median age (years)	48.8 ± 11.1	Shape rule (yes/no)	26/96
Immunohistochemistry		Clear boundary (yes/no)	50/66
ER (−/+/NA)	49/63/4	Aspect ratio (< 1/ > = 1)	6/110
PR (−/+/NA)	53/56/7	Echo uniformity (yes/no)	19/97
HER2 (−/+/NA)	36/58/22	Calcification (yes/no)	69/47
Ki67 (−/+/NA)	45/62/9	Intrafocal blood flow (yes/no)	79/37
P16 (−/+/NA)	29/45/42	Peripheral blood flow (yes/no)	32/84
P53(−/+/NA)	34/82/0	Catheter dilatation (yes/no)	9/107
Ultrasonic characteristics		lymph nodes (< 1/ > = 1)	93/23
Median size (cm)	2.6 ± 1.6	BI-RADS classification (3/4a/4b/4c/5/6)	12/31/30/23/9/11

### Radiomics analysis

**Fig. 4:**
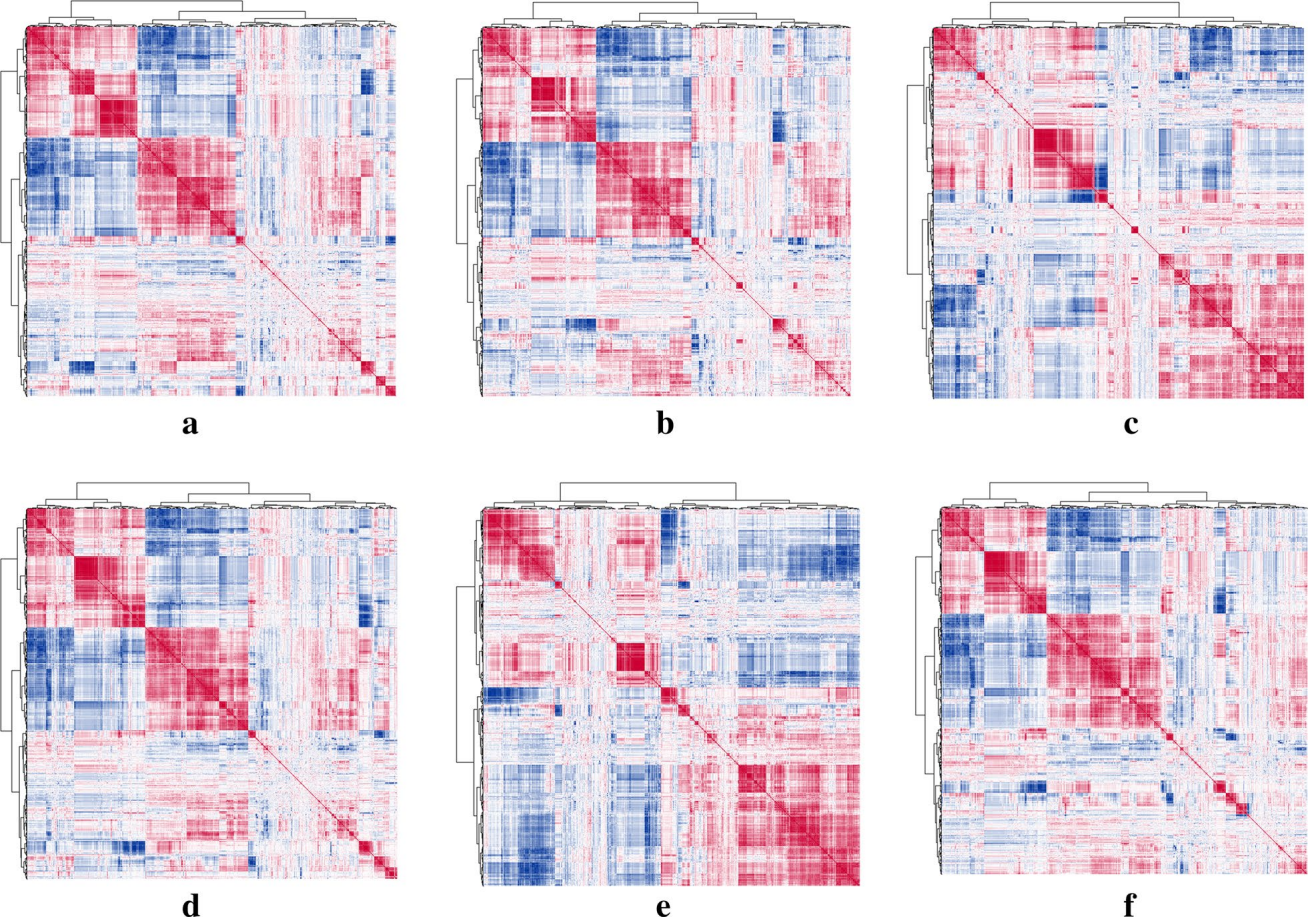
Correlation cluster analysis of 5234 radiomics features. The Pearson correlation test was used to analyze the correlation between features, and the "pheatmap" R software package was applied to draw heat maps. **a** ER; **b** PR; **c** HER2; **d** Ki67; **e** p16; **f** p53

**Fig. 5:**
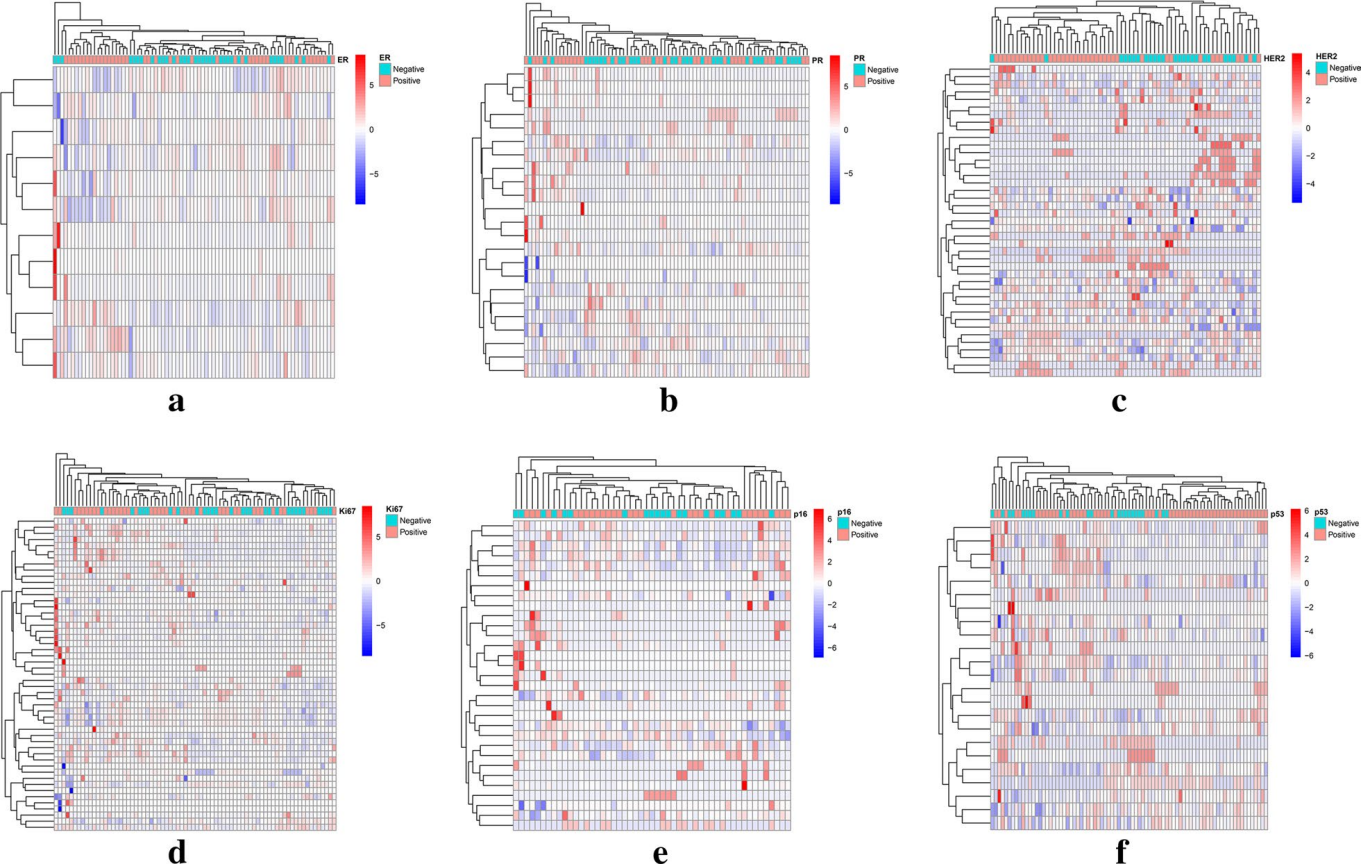
Important features for each molecular biomarkers. **a** ER; **b** PR; **c** HER2; **d** Ki67; **e** p16; **f** p53

The correlation clustering heatmaps among 5234 features of each molecular biomarkers (ER, PR, HER2, Ki67, p16, and p53) were shown in Fig. 4. A list of 18 feature importance methods were obtained, and the combination feature selection methods for optimal modeling results of each molecular biomarkers were as follows: Spearman0.75 + Statistical Test + RF, Spearman0.75 + Statistical Test + RF, Spearman0.75 + LASSO, Spearman0.75 + Statistical Test + RF, Spearman0.75 + Statistical Test + SVM-RFE, and Spearman0.85 + SVM-RFE. 12 features, 23 features, 41 features, 20 features, 31 features and 23 features were important for constructing prediction models. The heatmaps of the model features were presented in Fig. 5.

**Fig. 6:**
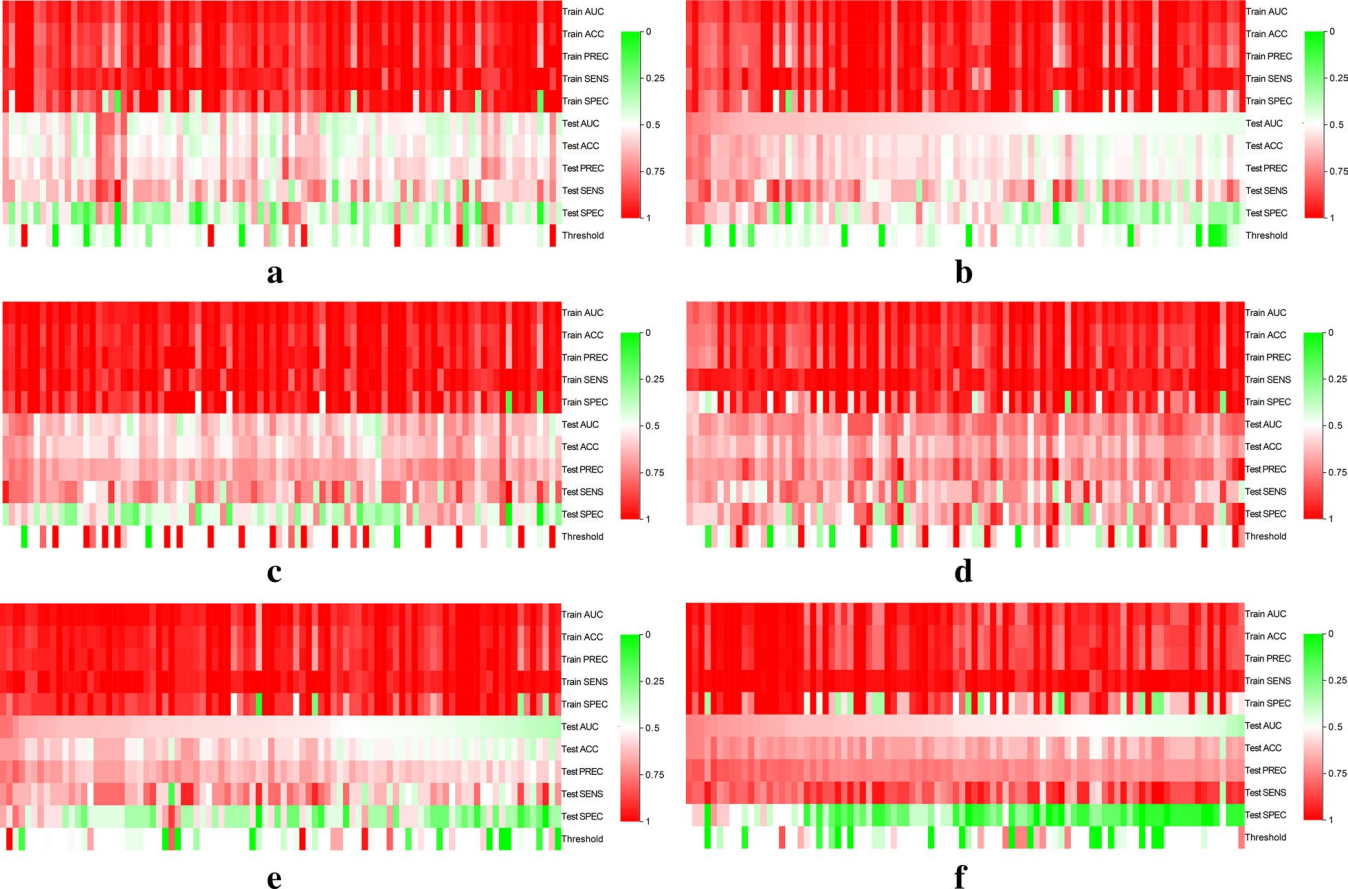
Heat maps of evaluation indicators for ninety radiomics prediction models. **a** ER; **b** PR; **c** HER2; **d **Ki67; **e** p16; **f** p53

**Fig. 7:**
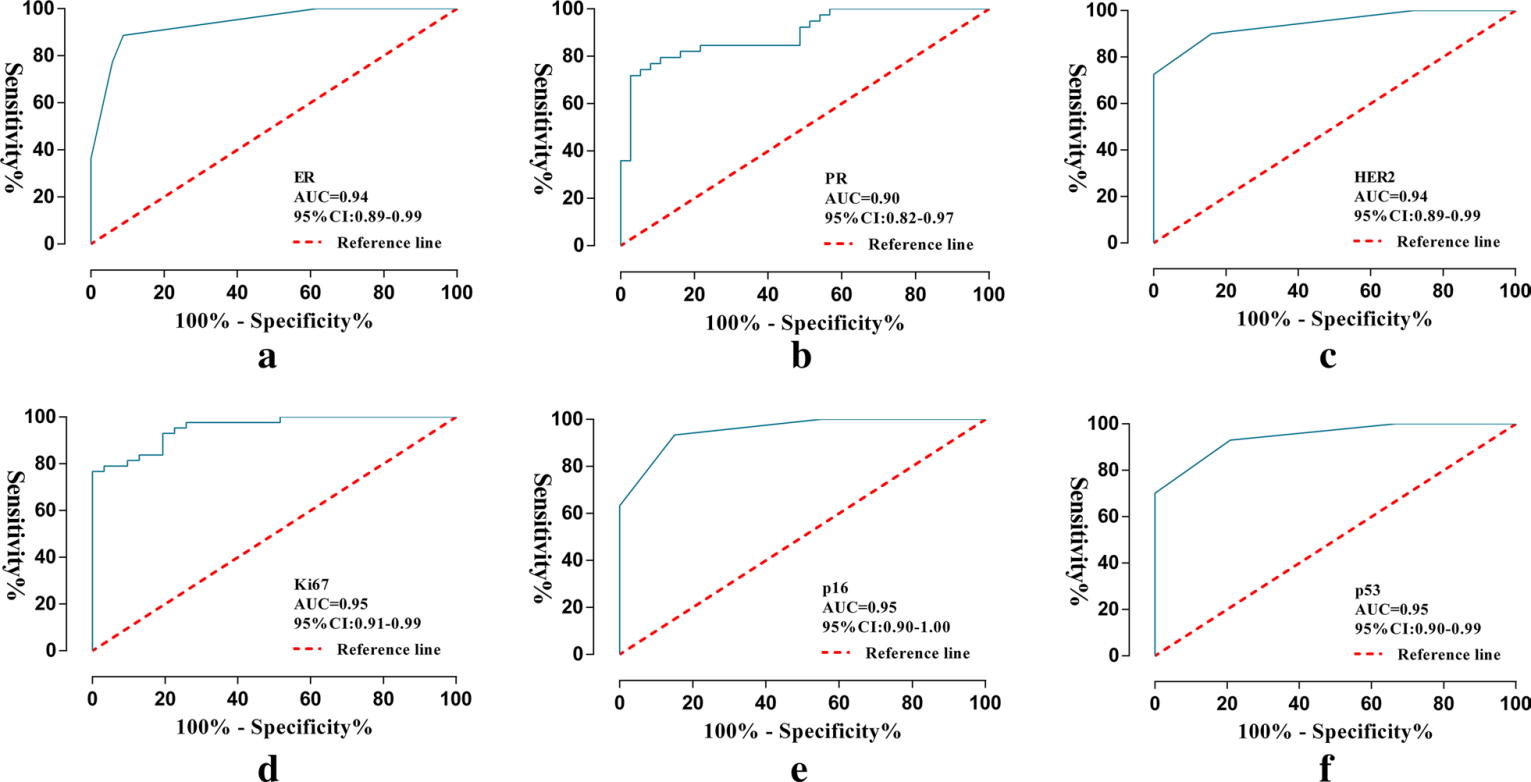
Performance of the radiomics models in the training set. **a** ER; **b** PR; **c** HER2; **d **Ki67; **e** p16; **f** p53

**Fig. 8:**
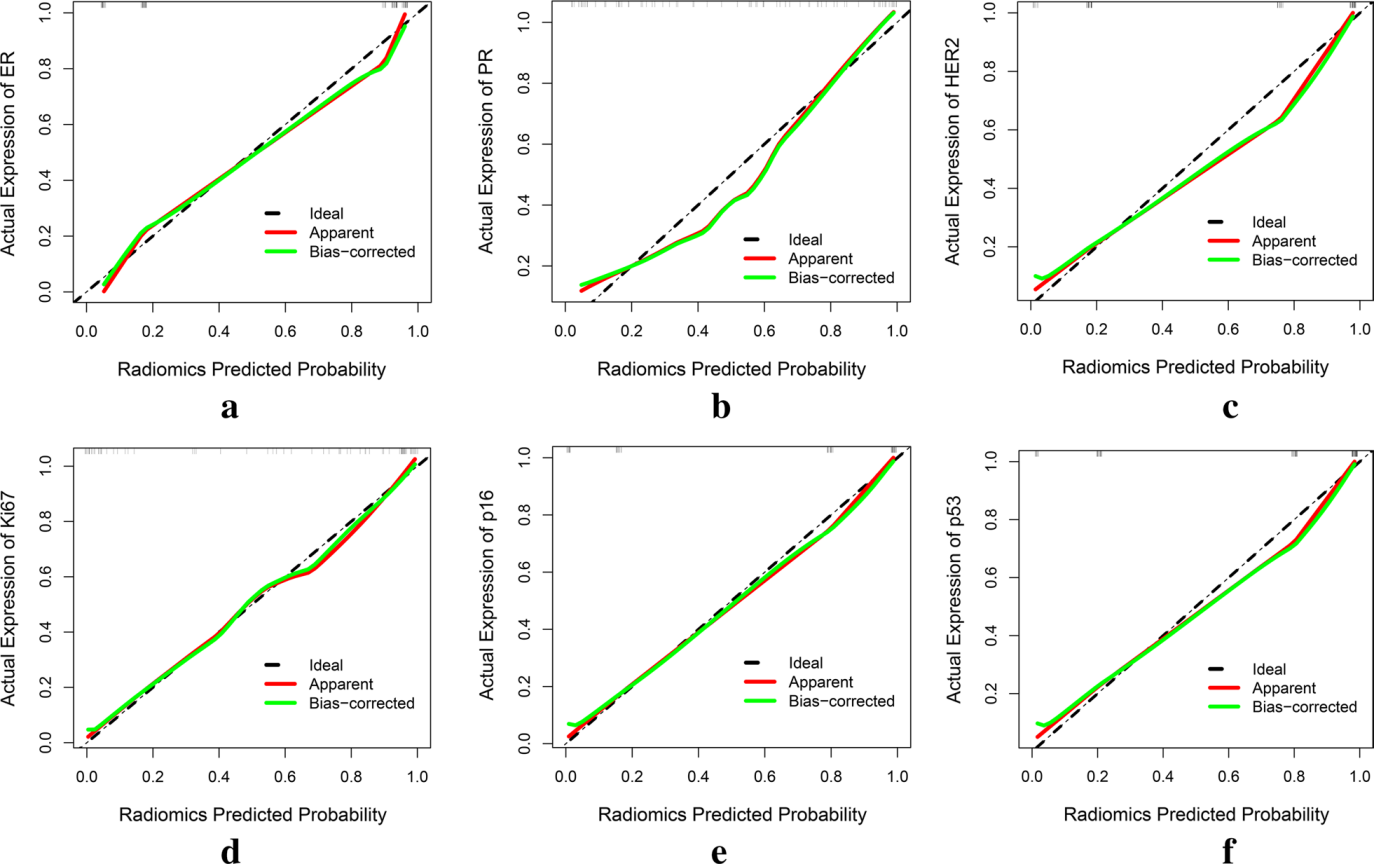
Calibration curves of the radiomics models in the training set. The oblique dashed line represents the perfect prediction of the ideal model. The solid line represents the performance of the radiomics model, and the dotted line near the diagonal indicates a better prediction. **a** ER; **b** PR; **c** HER2; **d **Ki67; **e** p16; **f** p53

**Fig. 9:**
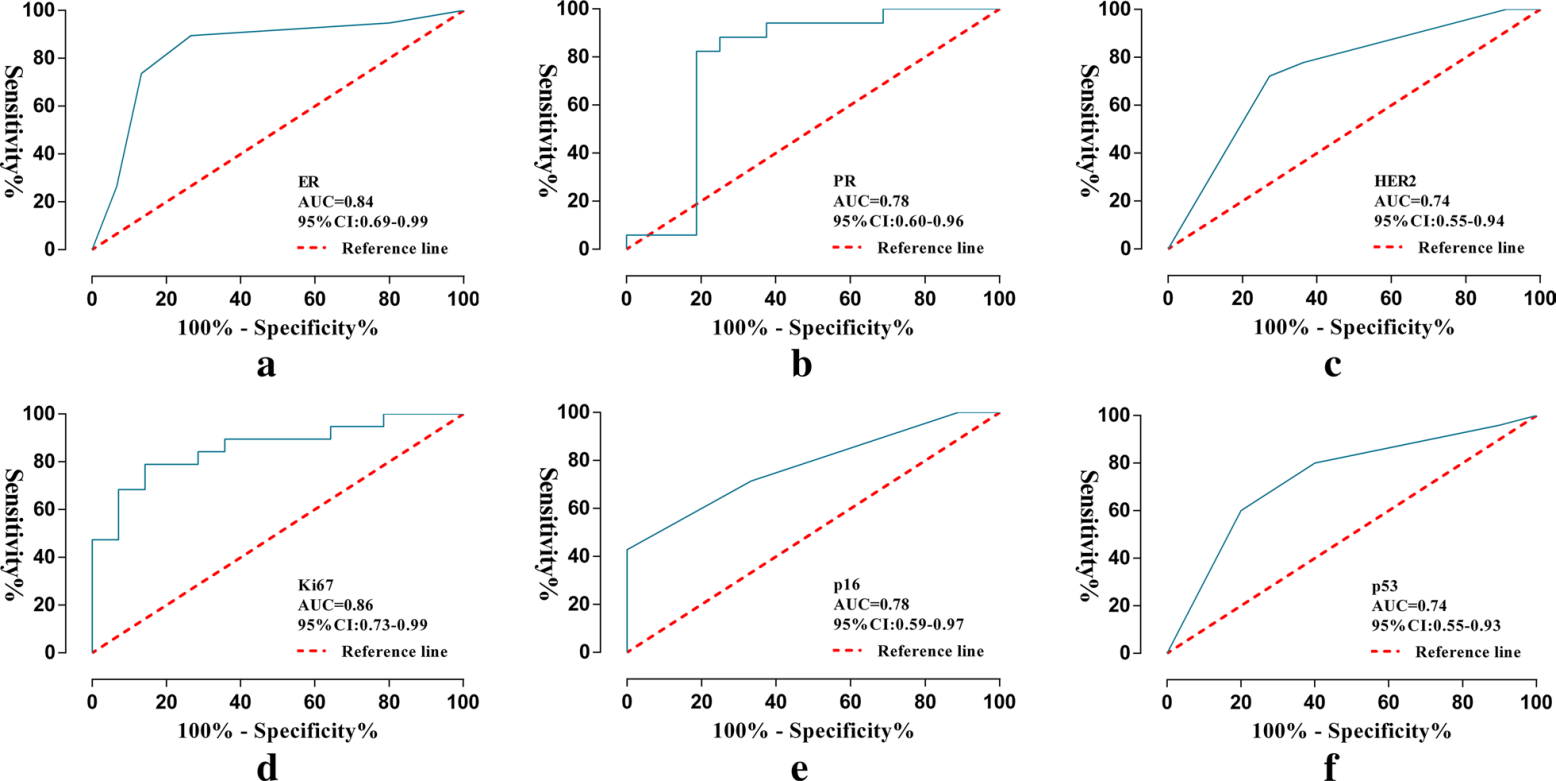
Performance of the radiomics models in the test set. **a** ER; **b** PR; **c** HER2; **d **Ki67; **e** p16; **f** p53

Ninety models were obtained by constructing prediction models with five classifiers, and the performance of the models were presented in Fig. 6 (Additional file 2). The optimal radiomics models were constructed by DT, SVM, KNN, SVM, KNN and KNN classifiers, respectively, and showed above moderate predictive performance in predicting the expression of molecular markers of DCIS (Table 2). Radiomics scores of training set and test set were significantly different in each molecular marker expression (training set, P < 0.001, test set, P < 0.05). The predictive performance of the radiomics models of each molecular biomarker in the training set: ER (AUC, 0.94, 95% confidence interval (CI) 0.89–0.99), PR (AUC, 0.90, 95% CI 0.83–0.97), HER2 (AUC, 0.94, 95% CI 0.89–0.99), Ki67 (AUC, 0.95, 95% CI 0.90–0.99), p16 (AUC, 0.96, 95% CI 0.91–1.00), p53 (AUC, 0.95, 95% CI 0.90–0.99), respectively (Fig. 7). The calibration curve of the prediction models in the training set confirmed the better consistency of the models (Fig. 8). The radiomics models showed predictive performance with AUC greater than 0.7 in the test set: ER (AUC, 0.84, 95% CI 0.68–0.99), PR (AUC, 0.78, 95% CI, 0.60–0.96), HER2 (AUC, 0.74, 95% CI 0.74–0.99), Ki67 (AUC, 0.86, 95% CI 0.67–0.97), p16 (AUC, 0.78, 95% CI 0.59–0.97), p53 (AUC, 0.74, 95% CI 0.55–0.93), respectively (Fig. 9).

**Table 2:** Evaluation of radiomics models in each DCIS molecular biomarkers

	Training set					Test set				
	AUC	ACC	PREC	Sn	Sp	AUC	ACC	PREC	Sn	Sp
ER	0.94	0.90	0.93	0.89	0.91	0.84	0.82	0.81	0.90	0.73
PR	0.90	0.84	0.89	0.80	0.89	0.78	0.76	0.80	0.71	0.8
HER2	0.94	0.88	0.90	0.90	0.84	0.74	0.72	0.78	0.78	0.64
Ki67	0.95	0.88	0.84	0.98	0.74	0.86	0.76	0.79	0.79	0.71
p16	0.96	0.90	0.90	0.94	0.85	0.78	0.70	0.77	0.71	0.67
p53	0.95	0.89	0.91	0.93	0.79	0.74	0.74	0.83	0.80	0.60

## Discussion

This study was the first non-invasive comprehensive analysis based on US-radiomics to predict the expression of molecular markers of DCIS. The investigators recruited only 116 patients with DCIS for this study, but it was exciting to see that the radiomics models showed more than moderate predictive performance in predicting molecular biomarker expression of DCIS.

DCIS is a malignant tumor with good prognosis, but it is heterogeneous in morphology and genetics. Before the imaging examination was performed, the diagnosis of DCIS was only due to the appearance of nipple discharge and/or palpable mass symptoms, which accounted for only 2% of DCIS detected. It showed that DCIS with hidden symptoms were easily missed [35]. With the screening of imaging technology (mammography, US and MRI), the detection rate of DCIS had gradually increased. This detection rate included symptomatic DCIS, and whether there was overdiagnosis in the detection of insidious DCIS was also a hot topic of controversy [36], Unfortunately, the diagnosis of DCIS marked women as at risk of invasive breast cancer, so women diagnosed with DCIS may suffer serious psychological distress, leading to the progression of DCIS [37]. In addition, the current treatment methods were also facing the controversy over the treatment of some patients [38]. Therefore, the main clinical challenge in DCIS has been to distinguish between patients who have a better chance of developing invasive cancer and require more treatment and those who are less likely to develop DCIS and need less or no treatment [39]. Immunohistochemical markers can explain the changes in the biological behaviors of tumors on the molecular level. More and more studies have pointed out the changes in molecular markers associated with the progression of DCIS to invasive cancer [40]. For example, Zhang GJ et al. [41] found that 79% of DCIS patients were positive for P53 when studying the occurrence and development of breast cancer. Davis et al. [42] demonstrated that high Ki67 expression was an independent predictor of postoperative recurrence in patients with DCIS. Cornfield DB had found a higher recurrence rate with PR > 3.5% using tree structure survival [43]. The results showed that the changes in the biological behavior of DCIS were closely related to the expression of molecular biomarkers.

About various imaging technologies, they also have application limitations [44]. Mammography is the main method of early breast cancer detection, but it is closely related to the density of the lesion and the possibility of covering the lesion [45]. However, Chinese women have dense breasts, so they had certain limitations in finding suspicious lesions in the dense tissues of breasts through mammography [46, 47]. The traditional mammography diagnosis method will cause trauma to the patient to a certain extent and reduce the patient's treatment compliance. Due to its high sensitivity to soft tissues, US can better show lesions in dense glands and has become the primary imaging method for Chinese women to screen and diagnose breast diseases. For non-mass DCIS, US is difficult to recognize [48]. Therefore, this retrospective study only examined mass DCIS, which is a limitation of the study. MRI has considerable advantages in detecting breast lesions, but its specificity is limited by several factors that affect image quality, such as magnetic field and gradient strength, coil performance, contrast agent efficacy and menstrual cycle [49].

Radiomics mainly studies the quantitative features that are related to biology in medical images. Radiomics features are considered the invisible tissue infrastructure components of the object to be imaged, which can serve as a valuable method for studying cancer by imaging, such as MRI. Radiomics can provide in vivo visualization and quantitative analysis of the imaging features of the whole imaging mass. Therefore, radiomics is a precision medical method for non-invasive diagnosis, evaluation of efficacy, biological behavior [50]. Currently, radiomics mainly relies machine learning algorithms to identify meaningful features of image training data set, and for further interpretation of the information and the optimization, so as to accurately predict the content of the research. An independent data set is applied to test the universality of the model and provides feedback for further optimization of the model [51]. To a certain extent, it improves the utilization of image information and enables differential diagnosis of diseases on more subtle levels that cannot be recognized by the naked eye.

Breast radiomics studies are mostly applied to the prediction of the molecular classification, lymph node metastasis and molecular markers of invasive ductal carcinoma. For example, Demircioglu A et al. [52] constructed radiomics models for predicting Ki67 expression in invasive breast cancer based on eight features extracted from MRI images, with an AUC of 0.81. Zhou et al. [53] explored the significance of MRI-radiomics models for predicting the expression of HER2 in patients with invasive breast cancer before surgery; the validation set AUC reached 0.81. There are few reports on DICS with radiomics. However, there are clinical challenges in the diagnosis and treatment of patients with DCIS. Tumor progression and treatment decisions are affected by multiple tumor molecular biomarkers, which require to comprehensively analyze and evaluate the molecular biomarkers of DCIS. To expand the application of radiomics in DCIS, the investigators carried out this study to assess the feasibility of molecular biomarkers of DCIS. The investigators believe that information obtained from multiple molecular biomarkers can help explain the underlying pathological process of DCIS.

In this study, the first highlight, the first comprehensive analysis of molecular markers of DCIS was conducted based on radiomics. Second highlight, there were thousands of radiomics features, including eight classifications: original feature can reflect the number of voxels in the images, intensity distribution, pixel pair frequency, image average gray value, size and shape of ROI (https://pyradiomics.readthedocs.io/en/latest/features.html); Ipris features capture nodular heterogeneity and differential growth patterns; CoLIAGe features can distinguish disease phenotypes that have similar morphologic appearances [54]; wavelets features represent most of the edge information in images; Shearlet features are better for processing high-dimensional signals; Gabors features extract the edge and gradient information of image and reflect the spatial frequency feature; PLBP, and WILBP: PLBP features are an oriented local texture descriptor that combines the phase congruency approach with the LBP. The third highlight of this study was to construct dozens of prediction models by combining multiple classifiers with multiple feature selection to select the optimal prediction results. RF and SVM-RFE had significant performance in feature selection of multiple molecular markers, KNN and SVM classification performed well too. Finally, through the verification of the test set, the prediction models all showed moderate performance.

There were also some shortcomings in our study. First, this retrospective study had the problem of small sample size. It was necessary to increase the sample size or multi-center cooperation to construct universal models. Second, when the radiologists manually delineated the ROIs, there were a certain degree of subjectivity to the contours of the lesions, which may lead to poor robustness of the models. In addition, the delineation process was done by only one radiologist. Third, the investigators only investigated the features extracted from the largest section, which could not represent the whole tumor. Due to the limitations of US, it was not possible to conduct three-dimensional studies similar to other imaging studies.

## Conclusion

The application of machine learning-based radiomics analysis provided a non-invasive method for predicting the expression of multiple molecular biomarkers in DCIS, with good prediction performance. This study also demonstrated the potential of radiomics in pathologic assessment and individualized precision therapy.

## Supplementary Information

**Additional file 1**. 5234 radiomics features matrix file.

**Additional file 2**. Modeling matrix file for molecular biomarkers.

## Data Availability

The datasets supporting the conclusions of this article were included within the article and its additional files.

## Abbreviations

DCIS: Ductal carcinoma in situ
ER: Estrogen receptor
PR: Progesterone receptor
HER2: Human epidermal growth factor receptor-2
AUC: Area under the curve
CI: Confidence interval
IHC: Immunohistochemistry
US: Ultrasound
MRI: Magnetic resonance imaging
BI-RADS: Breast imaging reporting and data system
ROI: Region of interest
CoLIAGe: Co-occurrence of local anisotropic gradient orientations
LBP: Local binary pattern
PLBP: Phased congruency-based local binary pattern
WILBP: Wavelet-based improved local binary pattern
GLCM: Gray level co-occurrence matrix
RLM: Run length matrices
LASSO: Least absolute shrinkage and selection operator
RF: Random forests
SVM-RFE: Support vector machine-recursive feature elimination
DT: Decision tree
KNN: K-nearest neighbors
LR: Logistics regression
NB: Naive Bayes
SVM: Support vector machine
ROC: Receiver operating curve
ACC: Accuracy
PREC: Precision
Sn: Sensitivity
Sp: Specificity
